# Placental growth factor measurements in the assessment of women with suspected Preeclampsia: A stratified analysis of the PARROT trial

**DOI:** 10.1016/j.preghy.2020.10.005

**Published:** 2021-03

**Authors:** Kate E. Duhig, Jenny E. Myers, Chris Gale, Joanna C. Girling, Kate Harding, Andrew Sharp, Nigel A.B. simpson, Derek Tuffnell, Paul T. Seed, Andrew H. Shennan, Lucy C. Chappell

**Affiliations:** a Department of Women and Children’s Health, School of Life Course Sciences, King’s College London (KED, PTS, AHS, LCC), United Kingdom; b The Division of Developmental Biology and Medicine, University of Manchester, United Kingdom; c Neonatal Medicine, School of Public Health, Faculty of Medicine, Chelsea and Westminster Campus, Imperial College London, United Kingdom; d West Middlesex University Hospital, Chelsea and Westminster Hospital NHS Foundation Trust, United Kingdom; e Guy’s and St Thomas’ NHS Foundation Trust, United Kingdom; f University of Liverpool and Liverpool Women’s Hospital, members of Liverpool Health Partners, United Kingdom; g Department of Women’s and Children’s Health, Faculty of Medicine and Health, University of Leeds, United Kingdom; h Bradford Institute for Health Research, United Kingdom

**Keywords:** Preeclampsia, PlGF, Hypertension in pregnancy, Diagnostic testing, PlGF, placental growth factor, NICE, National Institute of Health and Care Excellence

## Abstract

**Objective:**

Placental growth factor testing decreases time to recognition of preeclampsia and may reduce severe maternal adverse outcomes. This analysis aims to describe the clinical phenotype of women by PlGF concentration, and to determine the mechanism(s) underpinning the reduction in severe maternal adverse outcomes in the PARROT trial, in order to inform how PlGF testing may be optimally used within clinical management algorithms.

**Study design:**

This was a planned secondary analysis from the PARROT trial that compared revealed PlGF testing and management guidance with usual care in the assessment of women with suspected preterm preeclampsia.

**Main outcome measures:**

Maternal and perinatal outcomes following stratification of women by trial group, and measured PlGF concentration.

**Results:**

1006 women were included. PlGF < 100 pg/ml identified women with more marked hypertension, increased adverse maternal outcomes and preterm delivery rates, and higher rates of small for gestational age infants. There was a reduction in adverse maternal outcomes in women whose results were revealed when PlGF levels were 12–100 pg/ml compared to usual care (3.8% vs 6.9%; aOR 0.15(95% CI 0.03–0.92). There was no significant difference in gestation at delivery between concealed or revealed groups in any PlGF categories.

**Conclusion:**

Low PlGF concentrations are associated with severe preeclampsia. The reduction in severe adverse maternal outcomes may be mediated through quicker diagnosis and intensive surveillance, as recommended by the management algorithm for those at increased risk. PlGF is particularly beneficial in those who test 12–100 pg/ml, as these may be women with silent multi-organ disease who otherwise may go undetected.

## Highlights

• Low PlGF at presentation with suspected preeclampsia identifies a severe phenotype of disease.
• The use of PlGF testing leads to a reduction in severe maternal adverse outcomes.
• PlGF testing increases antenatal surveillance of women at risk of complications of preeclampsia.
• PlGF testing does not appear to cause an increase in preterm delivery rates.

## 1 Introduction

Preeclampsia complicates around 3% of singleton pregnancies, with hypertension affecting 10% of pregnant women [1], [2], [3]. Preeclampsia is associated with a high risk of pregnancy complications including iatrogenic preterm birth, maternal and perinatal morbidity, and perinatal mortality [4], [5], [6], [7].

The placenta plays a central role in the pathogenesis of preeclampsia. Studies of placentally-derived angiogenic factors, such as Placental Growth Factor (PlGF) and soluble fms-like tyrosine kinase-1 (sflt-1) have led to their development as adjuncts to diagnosis and prognosis [8], [9]. Evidence from prospective cohort studies has shown that angiogenic factors have good test performance for identifying preterm preeclampsia [10], [11]. These studies included women in whom angiogenic factor concentrations were concealed to carers. A recent randomised trial (PARROT) of 1023 women evaluated revealed PlGF measurement with a clinical management algorithm against usual care, forming one of the largest studies of angiogenic factors in the management of suspected preterm preeclampsia. In this trial there was a clinically important reduction in time to diagnosis of preeclampsia with a concurrent reduction seen in severe maternal adverse outcomes with revealed PlGF testing [12].

The aim of this secondary analysis of the PARROT Trial was to describe clinical phenotypes of pregnancies by measured PlGF concentration. The analysis also assesses how PlGF measurement may have impacted on clinical outcomes, to inform understanding of the mechanism of benefit. We sought to determine how PlGF testing may be optimally used within clinical management algorithms, by evaluating effect of PlGF testing across women categorised by their PlGF level. We focussed statistical testing on mechanistic questions related to how revealing an abnormal result might drive change in processes or pathways of care.

## 2 Methods

This was a planned secondary analysis of the PARROT trial, a multicentre stepped wedge cluster randomised controlled trial (ISRCTN 16842031), approved by the London South East Research Ethics Committee (15/LO/2058). Women were recruited from 11 centres with singleton pregnancies and a live fetus from 20^+0^ to 36^+^ [6] weeks’ gestation with suspected preeclampsia. Suspected preeclampsia was defined as new onset or worsening of existing hypertension, proteinuria, epigastric or right upper quadrant pain, headache with visual disturbances, altered maternal biochemistry or fetal growth restriction. Women were excluded if they had a confirmed diagnosis of preeclampsia at presentation. Randomisation was to intervention or control groups, and this occurred at cluster level, in a stepped wedge design.

Women in the control group received usual care following local hospital practice based on 2010 National Institute of Health and Care Excellence (NICE) guidance on the management of hypertension in pregnancy [13], with an additional blood sample taken for concealed PlGF testing. National guidance included treatment with oral labetalol, nifedipine or methyldopa if above the blood pressure target range, a blood pressure target of <150/100 mmHg on treatment, twice weekly blood pressure and urine checks in women with gestational hypertension, admission to hospital and delivery at 37 weeks’ gestation for those with preeclampsia. Women in the intervention group received revealed PlGF testing integrated into a standard clinical management algorithm based upon national guidance (Fig. S1). Women were individually consented to participation in the trial. A single PlGF blood sample was taken from each woman at presentation.

All blood samples were processed at each unit on a Triage (Alere, San Diego, CA, now Quidel Cardiovascular Inc., San Diego, CA) instrument. Strict logs of quality control assessment were kept. Staff performing the assay were trained by the trial team and followed a standard operating procedure. The test is CE-Marked and is approved for use in countries recognising the CE Mark. This clinical study was conducted in the European Union (U.K.) and investigated a clinical application approved under the product’s CE Mark.

### 2.1 Outcomes

Outcomes were collected until the primary postnatal discharge of the woman and infant pair from secondary care services. The primary outcome for the PARROT trial was the time from presentation with suspected preeclampsia to having a diagnosis of preeclampsia documented in the clinical notes. Maternal outcomes for this planned secondary analysis were a composite of severe maternal adverse outcomes as defined by the fullPIERS consensus [14], systolic blood pressure ≥ 160 mmHg, progression to severe preeclampsia (independently adjudicated) [15], placental abruption, mode and onset of delivery, use of antihypertensive medication, proportion of women reaching the diagnostic criteria (irrespective of clinical documentation) for preeclampsia [16]. These outcomes matched those used for the primary trial analysis.

Perinatal outcomes included gestation at delivery, preterm birth below 37 weeks’ gestation, birthweight and birthweight centile [17], a composite of severe perinatal adverse outcomes (number of babies with one or more of the following: intraventricular haemorrhage, seizures, retinopathy of prematurity, respiratory distress syndrome, bronchopulmonary dysplasia, necrotising enterocolitis stage 2 and 3 [18], stillbirth, early neonatal death and late neonatal death to 28 days), neonatal unit admission, perinatal death (stillbirths from 24 weeks’ gestation to deaths up to seven completed days after birth) and late neonatal death (deaths between 8 and 27 completed days of life).

### 2.2 Sample size

The sample size was determined for the main PARROT trial [12]. All women who participated in the trial who had a concealed or revealed PlGF result, and outcome data were included in this planned secondary analysis.

### 2.3 Statistical analysis

Women were stratified by their measured PlGF concentration into the following predetermined groups: PlGF ≥ 100 pg/ml – determined as ‘normal’; PlGF 12–99 pg/ml, equivalent to < 5th centile for gestation and determined as ‘low’; PlGF < 12 pg/ml, the lowest limit of detection for the assay and determined as ‘very low’.

These categorical groups were used based on the evidence that ‘low’ PlGF has a high diagnostic accuracy (0.96; 95% confidence interval, 0.89–0.99) and negative predictive value (0.98; 0.93–0.995) for determining preeclampsia requiring delivery in 14 days in prospective observational cohort studies [11], and ‘very low’ PlGF is the lowest limit of detection of the assay. We have previously reported that a fixed PlGF threshold of <100 pg/mL predicted preeclampsia requiring delivery within 14 days or before 37 weeks’ gestation (whichever was sooner) with sensitivity and negative predictive values similar to diagnostic accuracy estimates obtained by using a <5th centile cut-off. [11] The data were analysed according to their measured PlGF group. To describe clinical phenotype by measured PlGF level, demographic data are presented in the concealed testing group only. We compared how outcomes were influenced by trial arm in each subgroup in order to determine which groups of women benefited in our primary trial, and elucidate how this was achieved.

Outcomes were adjusted for centre and categorical time effects because of the trial design. Effects were estimated using multiple regression including terms for the intervention with fixed effects using dummy variables at each time in each centre. Centre was considered as a categorical variable and fitted as separate dummy variables for each centre. Calendar time was treated as a single categorical time variable. Continuous outcomes were assessed by linear regression. All binary outcomes were analysed using a binomial regression model with a log link. Test performance was evaluated using sensitivity, specificity, positive and negative predictive values and positive and negative likelihood ratios and area under the receiver operating characteristic curves. Mixed effects log-normal regression curves were generated for the proportion of women diagnosed relative to time from trial entry.

## 3 Results

**Fig. 1:**
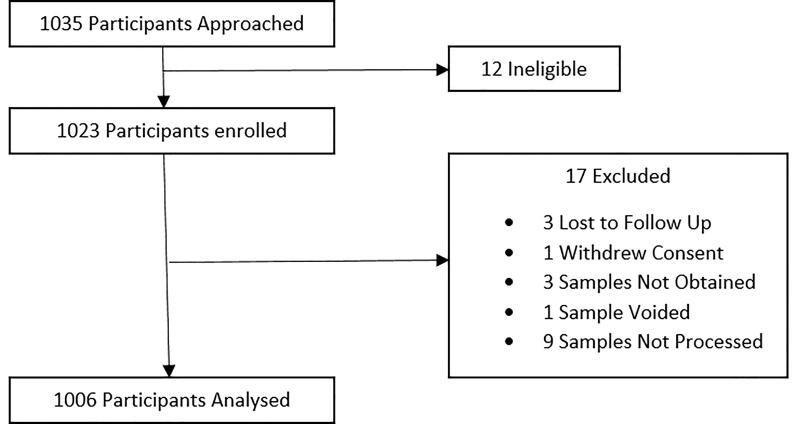
STROBE Diagram showing participant flow.

1006 women were included in this secondary analysis: 435 in the usual care group, and 571 in the revealed group (Fig. 1). The unequal size of the trial groups was due to the stepped wedge design, such that recruitment increased overall as the trial continued. There was no contamination between trial groups. Among the participants, 236 (23.5%) had PlGF < 12 pg/ml, 385 (38.3%) had PlGF 12–100 pg/ml, and 385 (38.3%) had PlGF > 100 pg/ml.

### 3.1 Clinical characteristics by PlGF category

In the concealed PlGF < 12 pg/ml category, 78.3% of women received a final diagnosis of preeclampsia. The mean highest systolic blood pressure in the 48 h prior to trial entry in all women was 150 (17) mmHg. The median gestation at delivery was 34.4 weeks, and 59% of the participants delivered within 14 days of enrolment to the trial. Of the babies born to women in the PlGF < 12 pg/ml category, 46% had a birthweight of <10th centile.

In the concealed PlGF 12–100 pg/ml category, 48.0% of women received a diagnosis of preeclampsia. The mean highest systolic blood pressure in the 48 h prior to trial entry was 144 (19) mmHg. The median gestation at delivery was 37.4 weeks, with 43% of participants delivered within 14 days of enrolment in the trial. 20% of the babies born to women in the PlGF 12–100 pg/ml category had a birthweight of <10th centile.

**Fig. 2:**
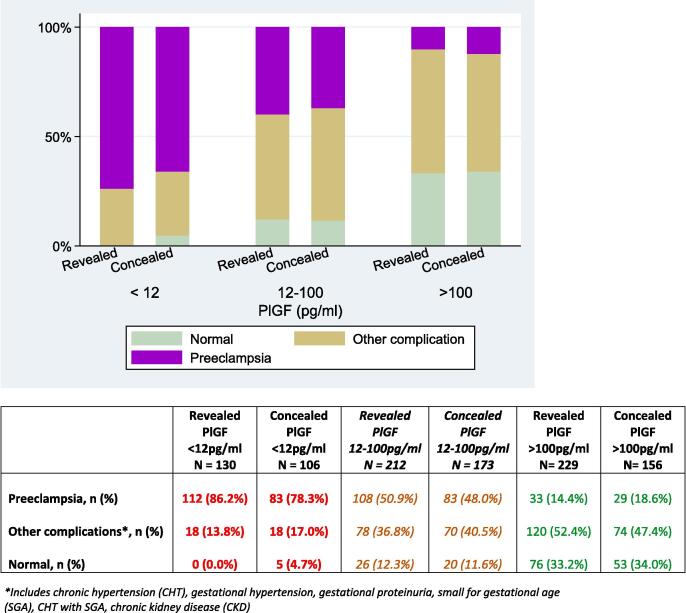
Final Diagnoses for Women in the PARROT Trial, stratified by PlGF category.

In the concealed PlGF > 100 pg/ml category, 18.6% of women received a final diagnosis of preeclampsia. The mean highest systolic blood pressure in the 48 h prior to trial entry was 136 (21) mmHg. The median gestation at delivery was 38.2 weeks, with 8% of participants delivered within 14 days of enrolment in the trial. 9% of the babies born to women in the PlGF > 100 pg/ml category had a birthweight of <10th centile. Further demographic details and corresponding values for the revealed group are presented in Table 1 and Table S1, and Fig. 2.

**Table 1:** Clinical characteristics.

	Revealed PlGF < 12 pg/ml N = 130	Concealed PlGF < 12 pg/ml N = 106	*Revealed PlGF 12*–*100 pg/ml* *N = 212*	*Concealed PlGF 12*–*100 pg/ml* *N = 173*	Revealed PlGF > 100 pg/ml N = 229	Concealed PlGF > 100 pg/ml N = 156
Age (years) Mean (SD)	**31.6 (6.0)**	**31.0 (6.1)**	*32.3 (6.0)*	*32.1 (5.7)*	31.7 (5.8)	31.2 (6.4)
Blood pressure at booking (mmHg)						
Systolic mean (SD)	**118 (15)**	**116 (15)**	*121 (14)*	*121 (16)*	120 (15)	121 (16)
Diastolic mean (SD)	**73 (11)**	**72 (11)**	*74 (10)*	*75 (12)*	74 (11)	75 (12)
Gestation at enrolment, weeks, median (IQR)	**32.3 (28.7,34.3)**	**34.1 (29.1,35.9)**	*34.6 (32.4,35.9)*	*35.1 (33.1,36.1)*	32.6 (29.1,34.9)	32.3 (28.9,34.7)
Primiparous (%)	**84 (64.6%)**	**62 (58.5%)**	*114 (53.8%)*	*85 (49.1%)*	114 (49.8%)	58 (37.2%)
Pre-existing chronic hypertension	**20 (15.4%)**	**15 (14.2%)**	*29 (13.7%)*	*28 (16.2%)*	37 (16.2%)	25 (16.0%)
Previous preeclampsia (%)	**24 (18.5%)**	**23 (21.7%)**	*42 (19.8%)*	*34 (19.7%)*	33 (14.5%)	33 (21.2%)
Highest blood pressure in 48 h prior to study entry (mmHg)						
Systolic mean (SD)	**153 (18)**	**150 (17)**	*146 (17)*	*144 (19)*	136 (20)	136 (21)
Diastolic mean (SD)	**97 (12)**	**97 (10)**	*93 (12)*	*93 (12)*	84 (13)	85 (14)
Time to diagnosis of preeclampsia (for those diagnosed) (days) Median (IQR) Effect size (time ratio (95%CI))	**1.0 (0.3, 4.5)**	**2.0 (0.3, 9.0) 0.17 (0.03 – 1.06)**	*2.0 (0.9, 8.70)*	*4.6 (1.0, 14.5) 0.66 (0.09*–*4.95)*	22.8 (8.4, 39.2)	30.3 (5.9, 65.1) 0.13 (0.16–1.07)
Number of women with clinician diagnosed preeclampsia n (%)	**96 (73.8%)**	**70 (66.0%)**	*84 (39.6%)*	*64 (37.0%)*	23 (10.0%)	19 (12.2%)
Severe preeclampsia (ACOG definition) n women (%)	**73 (56.2%)**	**49 (46.2%)**	*64 (30.2%)*	*49 (28.3%)*	18 (7.9%)	7 (4.5%)

	Revealed PlGF <12pg/ml N = 130	Concealed PlGF <12pg/ml N = 106	*Revealed PlGF 12-100pg/ml* *N* = *212*	*Concealed PlGF 12-100pg/ml* *N* = *173*	Revealed PlGF >100pg/ml N= 229	Concealed PlGF >100pg/ml N= 156
Preeclampsia, n (%)	**112 (86.2%)**	**83 (78.3%)**	*108 (50.9%)*	*83 (48.0%)*	33 (14.4%)	29 (18.6%)
Other complications*, n (%)	**18 (13.8%)**	**18 (17.0%)**	*78 (36.8%)*	*70 (40.5%)*	120 (52.4%)	74 (47.4%)
Normal, n (%)	**0 (0.0%)**	**5 (4.7%)**	*26 (12.3%)*	*20 (11.6%)*	76 (33.2%)	53 (34.0%)

* Preeclampsia includes those diagnosed with clinician diagnosed preeclampsia and those with a diagnosis of preeclampsia adjudicated by the clinical trial team. ** Includes chronic hypertension (CHT), gestational hypertension, gestational proteinuria, small for gestational age (SGA), CHT with SGA, chronic kidney disease (CKD).

### 3.2 Diagnosis of preeclampsia

The proportion of women with clinician diagnosed preeclampsia was not significantly different between the intervention (revealed) and usual care (concealed) in any of the PlGF categories (74% vs 66% for PlGF < 12 pg/ml, 40% vs 37% for PlGF 12–100 pg/ml, and 12% vs 10% for PlGF > 100 pg/ml).

Time to diagnosis of preeclampsia was lower in the revealed PlGF testing group (1.9 days) compared to usual care (4.1 days) across all three PlGF groups (time ratio 0.36, 95% CI 0.15–0.87; p = 0.027). Within PlGF categories, time to diagnosis in the revealed testing group vs. the concealed testing group was 1.0 vs 2.0 days (adjusted time ratio 0.17 (95% CI 0.03-1·06)) for PlGF < 12 pg/ml; 2.0 vs 4.6 days (adjusted time ratio 0.66 (95% CI 0.09–4.95)) for PlGF 12–100 pg/ml, and 22.8 vs 30.3 days (adjusted time ratio 0.13 (95% CI 0.16–1.07)) for PlGF > 100 pg/ml) (Table 2). Fig. S2 shows the mixed-effects lognormal regression curves with the proportion of women diagnosed by time from trial entry and differences in days (A), and weeks (B) with revealed PlGF testing in those women with PlGF < 12 pg/ml and PlGF 12–100 pg/ml, showing shortened time to diagnosis in both PlGF < 12 pg/ml or PlGF 12–100 pg/ml categories.

**Table 2:** Pregnancy outcomes.

	Revealed PlGF < 12 pg/ml N = 130	Concealed PlGF < 12 pg/ml N = 106	*Revealed PlGF 12*–*100 pg/ml* *N = 212*	*Concealed PlGF 12*–*100 pg/ml* *N = 173*	Revealed PlGF > 100 pg/ml N = 229	ConcealedPlGF > 100 pg/ml N = 156
Maternal adverse outcomes n of women (%) * aOR (95% CI)	**8 (6.2%)**	**6 (5.7%)** **0.87 (0.09 to 8.02)**	*8 (3.8%)*	*12 (6.9%)* *0.15 (0.03 to 0.92)*	6 (2.6%)	6 (3.8%) 0.29 (0.02 to 4.34)
Use of antenatal corticosteroids for fetal lung maturity n (%)	**98 (75.4%)**	**54 (50.9%)**	*67 (31.6%)*	*51 (29.5%)*	35 (15.3%)	22 (14.1%)
Those delivering < 35 weeks, % who got steroids in 7 days	**29/75 (38.6%)**	**6/38 (15.8%)**	*12/32 (37.5%)*	*5/19 (26.3%)*	3/6 (50.0%)	1/5 (20.0%)
Gestation at delivery, weeks						
Mean (SD)	**33.4 (3.13)**	**34.4 (3.72)**	*36.71 (2.48)*	*37.06 (2.04)*	38.30 (1.75)	38.23 (2.33)
Mean Difference (95% CI)		**−0.03 (-1.72 to 1.66)**		*−0.40 (-1.25 to 0.45)*		0.36 (-0.44 to 1.16)
Status at Birth n (%)						
Livebirth	**126 (96.9%)**	**102 (96.2%)**	*211 (99.5%)*	*171 (98.8%)*	229 (100.0%)	153 (98.1%)
Stillbirth	**4 (3.1%)**	**4 (3.8%)**	*1 (0.5%)*	*2 (1.2%)*	0 (0.0%)	2 (1.3%)
Miscarriage < 24 weeks	**0 (0.0%)**	**0 (0.0%)**	*0 (0.0%)*	*0 (0.0%)*	0 (0.0%	1 (0.6%)
Birthweight centile by INTERGROWTH						
Mean (SD) Mean Difference (95% CI)	**19.8 (22.4)**	**25.0 (28.8) 2.2 (-10.8 to 15.2)**	*41.5 (31.8)*	*44.1 (32.4) −2.2 (14.0 to 9.5)*	57.1 (31.5)	54.8 (30.9) 3.1 (-9.3 to 15.4)
Birthweight centile < 10^th^ aOR (95% CI)	**54 (41.5%)**	**48 (45.2%) 0.44 (0.15 to 1.27)**	*47 (22.2%)*	*35 (20.2%) 0.90 (0.33 to 2.48)*	23 (10.0%)	14 (9.0%) 1.85 (0.45 to 7.67)
Neonatal unit admission n (%) aOR (95% CI)	**93 (71.5%)**	**62 (58.5%) 2.37 (0.63**–**7.92)**	*73 (34.4%)*	*54 (31.2%) 2.37 (0.76*–*7.37)*	29 (12.7%)	27 (17.3%)
Perinatal adverse outcome, n of infants (%) ** aOR (95% CI)	**49 (37.7%)**	**27 (25.5%) 1.95 (0.64 to 6.00)**	*25 (11.8%)*	*23 (13.3%) 1.62 (0.45 to 5.89)*	12 (5.2%)	9 (5.8%) 3.84 (0.29 to 51.31)

*As defined by the fullPIERS consensus [14] (number of women with one or more of the following features; maternal death, eclampsia, Glasgow Coma Scale < 13, stroke, transient ischaemic attack, cortical blindness, posterior reversible encephalopathy, retinal detachment, positive inotropic support, infusion of third parenteral antihypertensive, myocardial ischaemia or infarction, blood oxygen saturations < 90%, 50% FiO_2_ for > 1 h, intubation (other than for caesarean section), pulmonary oedema, ionotropic support, transfusion of blood products, platelets < 50 × 10^9^ per litre, hepatic dysfunction, haematoma or rupture, severe acute kidney injury (creatinine > 150 µmol/L or > 200 µmol/L in chronic kidney disease, dialysis, placental abruption)).

**Number of babies with one or more of the following features: perinatal death, late neonatal death (8–27 completed days of life), necrotising enterocolitis (stage 2 or 3), respiratory distress syndrome, bronchopulmonary dysplasia, seizures, retinopathy of prematurity, intraventricular haemorrhage.

### 3.3 Maternal outcomes

Severe maternal adverse outcomes were less frequent with revealed PlGF testing than with usual care overall (22/573 (3.8%) versus 24/446 (5.4%); adjusted OR (aOR) 0.32, 95%CI 0.11–0.95). This was significant in women with PlGF 12–100 pg/ml (3.8% vs 6.9%; aOR 0.15 (95% CI 0.03–0.92)) (Table 2, Table S2). There were no significant differences seen in mean systolic or diastolic blood pressure, or the use of magnesium sulfate in the revealed compared to concealed groups in any of the PlGF categories (Table 1). There was an increase seen in the use of antihypertensive medication in the intervention groups versus the usual care group in women with PlGF < 12 pg/ml (83.1% vs 74.5%; aOR 3.85 (95% CI 1.03 to 8.28). There were no differences seen in the number of antenatal ultrasound scans, vaginal deliveries, or elective or emergency caesarean section rates between the intervention or usual care groups in any of the PlGF categories.

### 3.4 Time to delivery and steroid administration

**Fig. 3:**
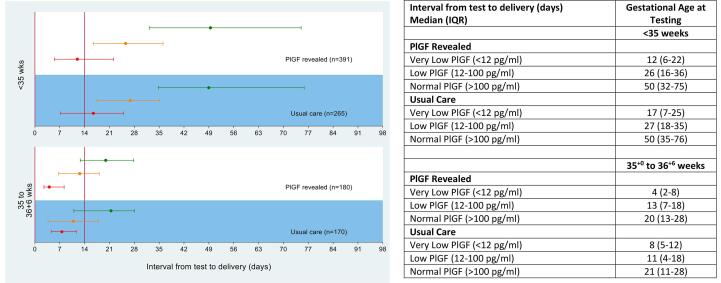
Time to Delivery (Median, IQR) stratified by PlGF concentration for all participants. Red line indicates PlGF < 12 pg/ml; orange line, PlGF 12–100 pg/ml; green line, PlGF > 100 pg/ml.

PlGF categorisation stratified by time to delivery is shown in Fig. 3; those with PlGF < 12 pg/ml, and < 100 pg/ml had consistently shorter times to delivery when compared to PlGF > 100 pg/ml.

In women who delivered < 35 weeks’ gestation, antenatal corticosteroids were given within the seven days prior to delivery in 39% (29/75) of the intervention group vs 16% (6/38) of the control group in women with PlGF < 12 pg/ml, and in 37.5% (12/32) and 26% (5/19) respectively in women with PlGF 12–100 pg/ml.).

### 3.5 Perinatal outcomes

There was no evidence of a difference significant difference in gestation at delivery, or perinatal adverse outcome rates with the intervention versus usual care in any of the PlGF categories (Table 2, Table S3). The difference in gestational age between the intervention and usual care in the < 12 pg/ml category was not significant (mean difference −0.03 weeks; −1.72 to 1.66). There were no significant differences in preterm delivery rates (<37 weeks’ gestation), or birthweight centiles between the intervention and usual care in any of the PlGF categories.

## 4 Discussion

In one of the largest studies of angiogenetic markers for the assessment of women with suspected preterm preeclampsia to date, we have confirmed that in a real-world setting, low and very low PlGF categories accurately identified women with a phenotype of more severe preeclampsia. Women with low and very low PGF concentrations have more marked hypertension, a greater number of adverse maternal outcomes, a shorter time to delivery interval and an increased need for preterm delivery, and higher rates of small for gestational age infants when compared with women with normal PlGF concentrations. Women with normal PlGF results have longer time to delivery intervals and rates of small for gestational age infants consistent with the general pregnant population.

PlGF testing does not lead to significantly more cases of preeclampsia being diagnosed, but consistently shortens the time it takes for a clinician to make a diagnosis across all three categories of PlGF. After adjustment for baseline characteristics, gestational age at delivery was not significantly different between the groups. PlGF testing did not appear to cause a significant difference in gestation at delivery by causing or preventing a non-indicated intervention through knowledge of the result.

Despite initial antenatal visit characteristics being very similar across all groups, women with very low PlGF concentrations had the most severe clinical phenotype of preeclampsia at entry to our study. However, whilst women with low PlGF concentrations appear to have an intermediate-risk phenotype of preeclampsia, they remain at increased risk of experiencing severe adverse outcomes compared to those with normal PlGF. One of the aims of stratification was to explore the mechanism(s) underlying the reduction seen in severe maternal adverse events with implementation of revealed PlGF testing. We found that the difference seen in the severe maternal adverse outcome composite was most marked in the PlGF 12–100 pg/ml group (aOR 0.15 (95% CI 0.03 to 0.92) and we anticipate that this may offer clinicians an opportunity to identify women at risk of developing severe preeclampsia complications, who may otherwise be considered at lower risk.

The improvement in clinical outcomes in this group may have been mediated by the use of the clinical management algorithm, which recommends increasing antenatal surveillance. Given that the proportion of women receiving a diagnosis is not increased with revealed PlGF, but that a diagnosis is made sooner after presentation, it would be reasonable to hypothesise that the mechanism for this reduction is mediated through increased surveillance and monitoring as recommended by the trial management algorithm. This may be particularly important in the group of women with PlGF 12–100 pg/ml who presented with clinical features of gestational hypertension but may also have had sub-clinical multi-organ disease features.

In this study we reiterated that a low or very low PlGF was not an indication for delivery in itself as highlighted by previous studies [19]. In the PlGF < 12 mg/ml group, women in the revealed group appear to deliver around one week earlier than those in the masked group (33.4 vs 34.4 weeks), but after pre-specified adjustment for baseline characteristics, this was not significant. It is also possible that within each PlGF category, those who needed earlier delivery were appropriately managed, and those clinically well were monitored, improving outcomes but not significantly changing overall preterm birth rates. However, whilst implementation of revealed PlGF testing does not significantly alter gestation at delivery between the two trial groups overall, we cannot exclude a difference in increasing preterm birth in the very low PlGF category that we were underpowered to demonstrate in this study. The results of the PREPARE study, which aims to determine if the use of sFlt/PlGF and a risk stratification algorithm reduces preterm delivery rates, are awaited [20].

Whilst the algorithm did not recommend routine admission for women with low or very low PlGF, and made no recommendations regarding steroid administration or timing of delivery which was left to the discretion of the treating clinicians, we hypothesise that low PlGF may have acted as an early warning sign of impending complications, giving clinicians the opportunity to act accordingly. The finding of the INSPIRE trial, in which women with suspected preeclampsia were individually randomised to revealed or concealed sFlt-1/PlGF testing demonstrated similar results, that the clinical use of PlGF/sFlt-1 testing enabled more accurate admission rates of high-risk patients without changing admission rates overall, and improved identification of those with preeclampsia [21].

There was a high prevalence of respiratory distress requiring NNU admission (nearly 30%) among babies with a PlGF < 12 pg/ml, but this was driven by gestational age at delivery. Of those women who delivered < 35 weeks’ gestation, revealed PlGF testing was associated with improvements in antenatal steroid administration within the seven days prior to delivery. Overall, 17% more women in the intervention (revealed) group received steroids within the seven days prior to delivery in those delivering < 35 weeks’ gestation, demonstrating that PlGF may be clinically useful in assisting with the timing of steroid administration.

Given that PARROT was a national, multicentre trial, we would anticipate that the prevalence of disease seen in the trial population would be similar in women presenting with suspected preterm preeclampsia to maternity triage settings throughout the UK. This would support the generalisability of these findings to the wider UK population.

A particular strength of our study is that these analyses focussed on identifying how the use of PlGF impacts on patient management pathways to influence important patient outcomes. This was a large multicentre study evaluating PlGF testing in a pragmatic way to achieve maximum external validity. The Patient Centred Outcomes Research Institute recommends the evaluation of process of care outcomes alongside morbidity outcomes in the evaluation of novel diagnostic tests [22]. It is known that effectiveness trials (i.e. in a real-world clinical care setting) can assess the overall performance of an intervention, but that it can be difficult to identify the exact processes that explain the effectiveness of an intervention, due their pragmatic nature [23]. Our cost effectiveness analysis has been previously reported. The resource use data showed that PlGF was overall cost saving, with an increase in antenatal inpatient costs for those with abnormal PlGF alongside a reduction in outpatient attendances in those with a normal result, suggesting improved risk stratification with PlGF testing [24]. As we did not undertake a more detailed process evaluation, the exact components of changes in the antenatal care pathways that contributed to the reduction in severe maternal adverse outcomes may remain unclear. However, this is balanced by the results being considerably more generalisable than if the trial had been undertaken with a very proscriptive management algorithm and multiple checkpoints such that the effect of the intervention might have required these additional components. Finding a significant effect size with a pragmatic algorithm suggests that clinicians found the intervention easy to integrate into their clinical care.

Stratification of the women in to six groups based on PlGF concentrations and treatment allocation has created smaller numbers in each comparison group, meaning we may be underpowered to demonstrate important differences in care. This was a planned secondary analysis of an existing trial dataset, and as such the interpretation of the results should be circumspect.

Previous comparative analyses of concealed versus revealed PlGF testing have demonstrated a reduction in perinatal deaths, but these analyses have been between two separate cohort studies with differing inclusion criteria, with a mixture of revealed and concealed testing [25]. This trial showed no difference in perinatal deaths with revealed testing; we anticipate that one reason for this is that 55% of the stillbirths in our trial occurred in pre-viable babies (<24 weeks’ gestation and <500 g), where intervention to influence outcome is limited. It may be that in order to prevent viable stillbirth, repeated PlGF testing is needed alongside ultrasound scanning as a means of disease monitoring, in order that interventions (including delivery) can be implemented in a timely manner in those babies at greatest risk of stillbirth. Further research is needed to determine the optimal frequency of repeat testing and to evaluate the impact on perinatal outcomes.

## 5 Conclusions

This analysis has shown that the use of revealed PlGF testing with appropriate clinical risk stratification particularly in those with low or very low PlGF, can prevent serious maternal adverse outcomes. PlGF is beneficial in identifying women with a phenotype indicative of placentally-driven disease, particularly those who test 12–100 pg/ml, in whom silent multi-organ disease may otherwise go undetected.

## Funding

This research was supported by grants from the 10.13039/501100000272National Institute for Health Research, Research for Patient Benefit Programme (PB-PG-0214-33054) and National Institute for Health Research Professorship (Chappell RP-2014–05-019). The views expressed in this publication are those of the author(s) and not necessarily those of the NHS, the National Institute for Health Research or the Department of Health. PS is funded in part by Tommy’s (registered charity number 1060508) and by the Collaboration for Leadership in Applied Health Research and Care South London (National Institute for Health Research). The funders had no involvement in the study design, collection and analysis of data, data interpretation report writing or the decision to submit the article for publication.

## Declaration of Competing Interest

The authors declare that they have no known competing financial interests or personal relationships that could have appeared to influence the work reported in this paper.
