# Supplementary material for: Placental growth factor measurements in the assessment of women with suspected Preeclampsia: A stratified analysis of the PARROT trial
Source: Pregnancy Hypertens. 2021 Mar;23:41–7. doi: 10.1016/j.preghy.2020.10.005 (PMC7909322; doi:10.1016/j.preghy.2020.10.005)

## **Supplementary Material for Placental Growth Factor Measurements in the Assessment of Women with Suspected Preeclampsia; a Stratified Analysis of the PARROT Trial.**

**Supplementary Tables**

**Table S1: Clinical Characteristics**

|  | **Revealed**  **PlGF <12pg/ml**  **N = 130** | **Concealed**  **PlGF <12pg/ml**  **N = 106** | ***Revealed***  ***PlGF 12-100pg/ml***  ***N = 212*** | ***Concealed***  ***PlGF 12-100pg/ml***  ***N = 173*** | **Revealed**  **PlGF >100pg/ml**  **N= 229** | **Concealed**  **PlGF >100pg/ml**  **N= 156** |
| --- | --- | --- | --- | --- | --- | --- |
| **Age (years)** Mean (SD) | **31.6 (6.0)** | **31.0 (6.1)** | *32.3 (6.0)* | *32.1 (5.7)* | 31.7 (5.8) | 31.2 (6.4) |
| **Body Mass Index (kg/m^2^)** Median (IQR) | **27.1 (24.1, 31.5)** | **29.0 (24.3,34.7)** | *27.9 (23.5, 33.7)* | *27.8 (24.3,33.3)* | 28.4 (24.0, 33.3) | 29.0 (23.9,34.5) |
| **Ethnicity n (%)**  White  Black  Asian  Mixed  Other (including Chinese) | **83 (63.8%)**  **11 (8.5%)**  **24 (18.5%)**  **3 (2.3%)**  **9 (6.9%)** | **65 (61.3%)**  **14 (13.2%)**  **20 (18.9%)**  **2 (1.9%)**  **5 (4.7%)** | *131 (62.1%)*  *31 (14.7%)*  *29 (13.7%)*  *4 (1.9%)*  *16 (7.6%)* | *106 (62.0%)*  *26 (15.2%)*  *22 (12.9%)*  *4 (2.3%)*  *13 (7.6%)* | 160 (71.2%)  33 (14.5%)  14 (6.2%)  6 (2.6%)  14 (6.2%) | 111 (71.4%)  22 (14.1%)  10 (6.4%)  5 (3.2%)  8 (5.1%) |
| **Parity n (%)**  0  1  2  >2 | **84 (64.6%)**  **22 (16.9%)**  **8 (6.2%)**  **16 (12.3%)** | **62 (58.5%)**  **21 (19.8%)**  **14 (13.2%)**  **9 (8.5%)** | *114 (53.8%)*  *50 (23.6%)*  *24 (11.3%) 24 (11.3%)* | *85 (49.1%)*  *49 (28.3%)*  *19 (11.0%)*  *20 (11.6%)* | 114 (49.8%)  61 (26.6%)  27 (11.8%)  27 (11.8%) | 58 (37.2%)  46 (29.5%)  30 (19.2%)  22 (14.1%) |
| **Previous preeclampsia (%)** | **24 (18.5%)** | **23 (21.7%)** | *42 (19.8%)* | *34 (19.7%)* | 33 (14.4%) | 33 (21.2%) |
| **Highest dipstick proteinuria in 48 hours prior to study entry n (%)**  None  Trace  +1  ≥+2 | **38 (29.2%)**  **9 (6.9%)**  **23 (17.7%)**  **60 (46.2%)** | **25 (23.6%)**  **10 (9.4%)**  **25 (23.6%)**  **46 (43.4%)** | *82 (38.7%)*  *20 (9.4%)*  *60 (28.3%)*  *50 (23.6%)* | *65 (37.6%)*  *27 (15.6%)*  *43 (24.9%)*  *38 (22.0%)* | 87 (39.2%)  30 (13.5%)  68 (30.6%)  37 (16.7%) | 77 (49.4%)  19 (12.2%)  37 (23.7%)  23 (14.7%) |
| **Pre-existing chronic hypertension** | **20 (15.4%)** | **15 (14.2%)** | *29 (13.7%)* | *28 (16.2%)* | 37 (16.2%) | 25 (16.0%) |
| **Blood pressure at booking (mmHg)**  Systolic mean (SD)  Diastolic mean (SD) | **118 (15)**  **73 (11)** | **116 (15)**  **72 (11)** | *121 (14)*  *74 (10)* | *121 (16)*  *75 (12)* | 120 (15)  74 (11) | 121 (16)  75 (12) |
| **Prophylactic aspirin prescribed n (%)** | **57 (43.8%)** | **34 (32.1%)** | *83 (39.2%)* | *72 (41.6%)* | 93 (41.2%) | 66 (42.3%) |
| **Presenting signs and symptoms (non-exclusive) n (%)**  New onset hypertension  Worsening of existing hypertension  New onset proteinuria  Epigastric/right upper quadrant pain  Neurological Symptoms  Suspected fetal growth restriction  Reduced fetal movements | **87 (66.9%)**  **27 (20.8%)**  **60 (46.2%)**  **9 (6.9%)**  **40 (30.8%)**  **45 (34.6%)**  **0 (0.0%)** | **65 (61.3%)**  **18 (17.0%)**  **46 (43.4%)**  **11 (10.4%)**  **31 (29.2%)**  **22 (20.8%)**  **0 (0.0%)** | *127 (59.9%)*  *40 (18.9%)*  *50 (23.6%)*  *19 (9.0%)*  *59 (27.8%)*  *32 (15.1%)*  *0 (0.0%)* | *85 (49.1%)*  *40 (23.1%)*  *38 (22.0%)*  *14 (8.1%)*  *52 (30.1%)*  *23 (13.3%)*  *0 (0.0%)* | 84/229 (36.7%)  32/229 (14.0%)  37/222 (16.7%)  18/229 (7.9%)  87/229 (38.0%)  25/229 (10.9%)  6/229 (2.6%) | 58/156 (37.2%)  21/156 (13.5%)  23/156 (14.7%)  21/156 (13.5%)  62/156 (39.7%)  15/156 (9.6%)  2/156 (1.3%) |
| **Primary Diagnosis, n (%)**  Clinically diagnosed preeclampsia  Clinically diagnosed superimposed preeclampsia  **All other diagnoses**  Gestational or chronic hypertension  Gestational Proteinuria  Isolated SGA/IUGR  Pre-existing kidney disease  Normal  Preeclampsia by adjudication | **84 (64.6%)**  **12 (9.2%)**  **13 (10.0%)**  **1 (0.8%)**  **3 (2.3%)**  **1 (0.8%)**  **0 (0.0%)**  **16 (12.3%)** | **61 (57.6%)**  **9 (8.5%)**  **11 (10.4%)**  **1 (0.9%)**  **6 (5.7%)**  **0 (0.0%)**  **5 (4.7%)**  **13 (12.7%)** | *71 (33.4%)*  *13 (6.1%)*  *57 (26.8%)*  *8 (3.8%)*  *13 (6.1%)*  *0 (0.0%)*  *26 (12.3%)*  *24 (11.3%)* | *50 (28.9%)*  *14 (8.1%)*  *53 (30.6%)*  *4 (2.3%)*  *13 (7.5%)*  *0 (0.0%)*  *20 (11.6%)*  *19 (11.0%)* | 18 (7.9%)  5 (2.2%)  78 (34.6%)  20 (8.7%)  16 (7.0%)  6 (2.6%)  76 (33.2%)  10 (4.4%) | 13 (8.3%)  6 (3.8%)  46 (29.5%)  15 (9.6%)  9 (5.8%)  4 (2.6%)  53 (34.0%)  10 (6.4%) |
| **Use of antihypertensives (%)**  Effect Size (95% CI) | **108 (83.1%)** | **79 (74.5%)**  **3.85 (1.03 to 8.28)** | *136 (64.2%)* | *122 (70.5%)*  *0.57 (0.25 to 1.31)* | 101 (44.1%) | 64 (41.0%)  0.70 (0.30 to 1.61) |
| **Use of magnesium sulfate n (%)**  Effect Size (95% CI) | **47 (36.2%)** | **39 (36.9%)**  **1.35 (0.45 to 4.06)** | *19 (9.0%)* | *19 (11.1%)*  *1.02 (0.27 to 3.84)* | 6 (2.6%) | 5 (3.2%)  - |
| **Use of antenatal corticosteroids for fetal lung maturity n (%)** | **98 (75.4%)** | **54 (50.9%)** | *67 (31.6%)* | *51 (29.5%)* | 35 (15.3%) | 22 (14.1%) |
| **Those delivering <35 weeks, % who got steroids in 7 days** | **29/75 (39%)** | **6/38 (16%)** | *12/32 (37.5%)* | *5/19 (26%)* | 3/6 (50%) | 1/5 (20%) |
| **Mode of delivery n (%)**  Spontaneous vaginal cephalic  Assisted vaginal  Caesarean section  Pre-labour Caesarean  In labour Caesarean | **27 (20.8%)**  **5 (3.8%)**  **52 (40.0%)**  **46 (35.4%)** | **25 (23.6%)**  **4 (3.9%)**  **38 (35.8%)**  **39 (37.9%)** | *78 (36.8%)*  *18 (8.5%)*  *56 (26.4%)*  *60 (28.3%)* | *76 (43.9%)*  *15 (8.7%)*  *48 (27.7%)*  *34 (19.7%)* | 105 (45.9%)  19 (8.2%)  62 (27.1%)  43 (18.8%) | 78 (50.0%)  20 (12.8%)  39 (25.0%)  19 (12.2%) |
| **Status at Birth n (%)**  Livebirth  Stillbirth  Miscarriage <24 weeks | **126 (96.9%)**  **4 (3.1%)**  **0 (0.0%)** | **102 (96.2%)**  **4 (3.8%)**  **0 (0.0%)** | *211 (99.5%)*  *1 (0.5%)*  *0 (0.0%)* | *171 (98.8%)*  *2 (1.2%)*  *0 (0.0%)* | 227 (100.0%)  0 (0.0%)  0 (0.0% | 153 (98.1%)  2 (1.3%)  1 (0.6%) |
| **Stillbirth Category, n**  Pre-Viable*  Viable (dysmorphism)  Viable (no dysmorphism) | **3**  **0**  **1** | **2**  **1**  **1** | *1*  *0*  *0* | *0*  *0*  *2* | 0  0  0 | 0  2  0 |
| **Early Neonatal death (%)** | **0 (0.0%)** | **0 (0.0%)** | *0 (0.0%)* | *0 (0.0%)* | 0 (0.0%) | 0 (0.0%) |
| **Late neonatal death (%)** | **1 (0.8%)** | **1 (1.0%)** | *2 (0.9%)* | *0 (0.0%)* | 0 (0.0%) | 0 (0.0%) |
| **Death before discharge (%)** | **1 (0.8%)** | **2 (1.9%)** | *3 (1.4%)* | *0 (0.0%)* | 0 (0.0%) | 1 (0.6%) |
| **Baby sex**  Male  Female | **61 (46.9%)**  **69 (53.1%)** | **62 (58.5%)**  **44 (41.5%)** | *105 (49.5%)*  *107 (50.5%)* | *88 (50.9%)*  *85 (49.1%)* | 107 (46.9%)  121 (53.1%) | 83 (53.2%)  73 (46.8%) |
| **Birthweight (grams)**  Mean (SD)  Mean Difference (95% CI) | **1720 (682)** | **2044 (956)**  **-36 (-441 to 369)** | *2671 (757)* | *2766 (692)*  *-140 (-404 to 124)* | 3176 (636) | 3127 (645)  100 (-154 to 353) |
| **Birthweight centile <3^rd^**  Effect size (95% CI) | **30 (23.1%)** | **24 (22.9%)**  **0.40 (0.11 to 1.41)** | *21 (10.0%)* | *16 (9.2%)*  *2.22 (0.51 to 9.60)* | 7 (3.1%) | 3 (1.9%) |
| **Apgar at 5 minutes**  Median (IQR) | **9 (9, 10)** | **9 (9, 10)** | *10 (9, 10)* | *10 (9, 10)* | 10 (9, 10) | 10 (9,10) |
| **Apgar <7 at 5 minutes n (%)**  Effect size (95% CI) | **14 (11.0%)** | **7 (7.1%)**  **0.48 (0.63 to 3.57)** | *12 (5.7%)* | *10 (6.1%)*  *0.66 (0.13 to 3.36)* | 8 (3.5%) | 5 (3.3%)  6.36 (0.41 to 98.6) |

**Table S2: Composite of Severe Maternal Adverse Outcomes Stratified by PlGF Level**

|  | **Revealed**  **PlGF <12pg/ml**  **N = 130** | **Concealed**  **PlGF <12pg/ml**  **N = 106** | **Revealed**  **PlGF 12-100pg/ml**  **N = 212** | **Concealed**  **PlGF 12-100pg/ml**  **N = 173** | **Revealed**  **PlGF >100pg/ml**  **N= 229** | **Concealed**  **PlGF >100pg/ml**  **N= 156** |
| --- | --- | --- | --- | --- | --- | --- |
| **Maternal adverse outcomes n of women (%) ^a^**  Effect Size (95% CI) | **8 (6.2%)** | **6 (5.7%)**  **0.87 (0.09 to 8.02)** | *8 (3.8%)* | *12 (6.9%)*  *0.15 (0.03 to 0.92)* | 6 (2.6%) | 6 (3.8%)  0.29 (0.02 to 4.34) |
| **Maternal death n (%)** | **0 (0.0%)** | **0 (0.0%)** | *0 (0.0%)* | *0 (0.0%)* | 0 (0.0%) | 0 (0.0%) |
| Central nervous system n (%)  GCS < 13 (Eclampsia)  Stroke  Hypertensive encephalopathy  Posterior reversible encephalopathy  Cortical blindness or retinal detachment | **0 (0.0%)**  **0 (0.0%)**  **0 (0.0%)**  **0 (0.0%)**  **0 (0.0%)** | **0 (0.0%)**  **0 (0.0%)**  **0 (0.0%)**  **0 (0.0%)**  **0 (0.0%)** | *0 (0.0%)*  *0 (0.0%)*  *0 (0.0%)*  *0 (0.0%)*  *0 (0.0%)* | *2 (1.2%)*  *2 (1.2%)*  *0 (0.0%)*  *0 (0.0%)*  *0 (0.0%)* | 0 (0.0%)  0 (0.0%)  0 (0.0%)  0 (0.0%)  0 (0.0%) | 0 (0.0%)  0 (0.0%)  0 (0.0%)  0 (0.0%)  0 (0.0%) |
| Cardiovascular/ respiratory n (%)  Myocardial infarction  Intubation (other than for caesarean section)  Pulmonary oedema  Inotropic support  SpO2 <90%  50% FiO2 >1 hour  Infusion of third parenteral antihypertensive | **0 (0.0%)**  **0 (0.0%)**  **2 (1.5%)**  **0 (0.0%)**  **0 (0.0%)**  **0 (0.0%)**  **0 (0.0%)** | **0 (0.0%)**  **0 (0.0%)**  **0 (0.0%)**  **0 (0.0%)**  **0 (0.0%)**  **0 (0.0%)**  **0 (0.0%)** | *0 (0.0%)*  *0 (0.0%)*  *0 (0.0%)*  *0 (0.0%)*  *1 (0.5%)*  *0 (0.0%)*  *1 (0.5%)* | *1 (0.6%)*  *1 (0.6%)*  *0 (0.0%)*  *0 (0.0%)*  *1 (0.6%)*  *0 (0.0%)*  *1 (0.6%)* | 0 (0.0%)  0 (0.0%)  0 (0.0%)  0 (0.0%)  0 (0.0%)  0 (0.0%)  0 (0.0%) | 0 (0.0%)  0 (0.0%)  0 (0.0%)  0 (0.0%)  0 (0.0%)  0 (0.0%)  1 (0.6%) |
| Haematological n (%)  Platelets <50×10⁹/L  Disseminated intravascular coagulation  Thrombotic thrombocytopenic purpura/ haemolytic uraemic syndrome | **2 (1.5%)**  **0 (0.0%)**  **0 (0.0%)** | **2 (2.0%)**  **0 (0.0%)**  **0 (0.0%)** | *1 (0.5%)*  *0 (0.0%)*  *0 (0.0%)* | *2 (1.2%)*  *0 (0.0%)*  *0 (0.0%)* | 1 (0.5%)  0 (0.0%)  0 (0.0%) | 0 (0.0%)  0 (0.0%)  0 (0.0%) |
| Hepatic n (%)  Haematoma or rupture  Acute fatty liver of pregnancy  Liver failure | **0 (0.0%)**  **1 (0.8%)**  **0 (0.0%)** | **0 (0.0%)**  **0 (0.0%)**  **0 (0.0%)** | *0 (0.0%)*  *0 (0.0%)*  *0 (0.0%)* | *0 (0.0%)*  *0 (0.0%)*  *0 (0.0%)* | 0 (0.0%)  0 (0.0%)  0 (0.0%) | 0 (0.0%)  0 (0.0%)  0 (0.0%) |
| Renal  Severe AKI (creatinine >150, or >200 in CKD) n (%)  Dialysis | **1 (0.8%)**  **0 (0.0%)** | **1 (1.0%)**  **0 (0.0%)** | *2 (0.9%)*  *0 (0.0%)* | *2 (1.2%)*  *0 (0.0%)* | 4 (1.7%)  0 (0.0%) | 4 (2.6%)  1 (0.6%) |
| Other adverse events  Transfusion of blood products | **4 (3.1%)** | **5 (4.7%)** | *4 (1.9%)* | *6 (3.5%)* | 1 (0.4%) | 3 (1.9%) |

^a^ As defined by the fullPIERS consensus (number of women with one or more of the following features; maternal death, eclampsia, Glasgow Coma Scale <13, stroke, transient ischaemic attack, cortical blindness, posterior reversible encephalopathy, retinal detachment, positive inotropic support, infusion of third parenteral antihypertensive, myocardial ischaemia or infarction, blood oxygen saturations <90%, 50% FiO_2_ for > 1 hour, intubation (other than for caesarean section), pulmonary oedema, ionotropic support, transfusion of blood products, platelets <50x10^9^ per litre, hepatic dysfunction, haematoma or rupture, severe acute kidney injury (creatinine >150 µmol/L or >200 µmol/L in chronic kidney disease, dialysis, placental abruption)).

**Table S3: Composite of Severe Perinatal Adverse Outcomes Stratified by PlGF Level**

|  | **Revealed**  **PlGF <12pg/ml**  **N = 130** | **Concealed**  **PlGF <12pg/ml**  **N = 106** | **Revealed**  **PlGF 12-100pg/ml**  **N = 212** | **Concealed**  **PlGF 12-100pg/ml**  **N = 173** | **Revealed**  **PlGF >100pg/ml**  **N= 229** | **Concealed**  **PlGF >100pg/ml**  **N= 156** |
| --- | --- | --- | --- | --- | --- | --- |
| **Perinatal adverse outcome, n of infants (%)**  OR (95% CI) | **49 (37.7%)** | **27 (25.5%)**  **1.95 (0.64 to 6.00)** | *25 (11.8%)* | *23 (13.5%)*  *1.62 (0.45 to 5.89)* | 12 (5.2%) | 9 (5.8%)  3.84 (0.29 to 51.31) |
| Perinatal adverse outcomes (composite; non-exclusive) n (%)  **Central nervous system:**  Intraventricular haemorrhage  Seizure  Retinopathy of prematurity  **Respiratory:**  Respiratory distress syndrome  Bronchopulmonary dysplasia  **Gastrointestinal:**  Necrotising enterocolitis (stage 2 or 3) | **5 (4.0%)**  **0 (0.0%)**  **44 (34.9%)**  **4 (3.2%)**  **4 (3.2%)** | **7 (6.7%)**  **0 (0.0%)**  **24 (22.9%)**  **2 (1.9%)**  **5 (4.8%)** | *1 (0.5%)*  *0 (0.0%)*  *25 (11.8%)*  *1 (0.5%)*  *3 (1.4%)* | *2 (1.2%)*  *1 (0.6%)*  *21 (12.3%)*  *1 (0.6%)*  *1 (0.6%)* | 1 (0.4%)  0 (0.0%)  10 (4.4%)  0 (0.0%)  0 (0.0%) | 1 (0.6%)  1 (0.6%)  8 (3.8%)  0 (0.0%)  1 (0.6%) |
| **Status at Birth n (%)**  Stillbirth | **4 (3.1%)** | **4 (3.8%)** | *1 (0.5%)* | *2 (1.2%)* | 0 (0.0%) | 2 (1.3%) |

**Figure S1: Clinical Management Algorithm**


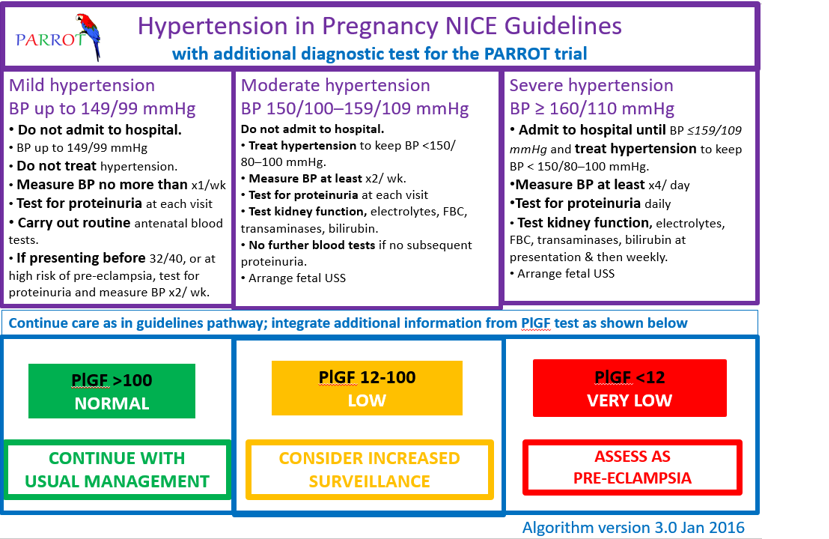


**Figure S2**

**Proportion of women diagnosed with preeclampsia over time; mixed-effects lognormal regression curves showing proportion of women diagnosed by time from trial entry showing differences in days (A), and weeks (B) with revealed PlGF testing. Red lines represent PlGF <12pg/ml, orange lines represent PlGF 12-100pg/ml.**


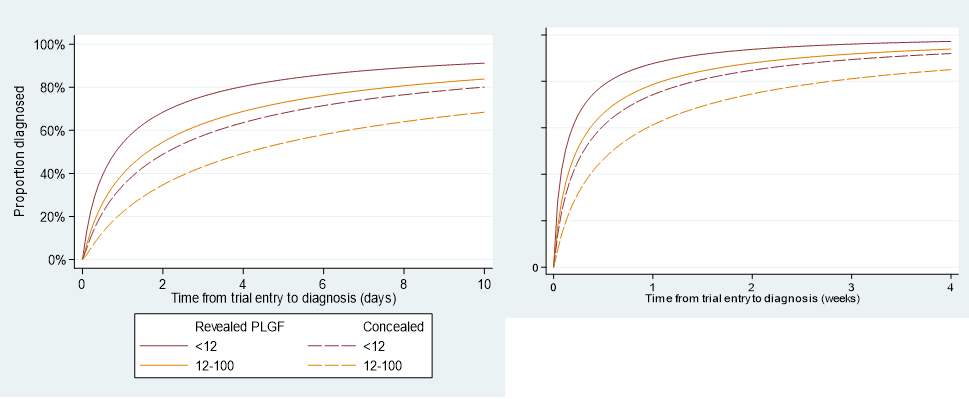

Supplement: Supplementary data 1 [file mmc1.docx]
